# Hippo信号通路在肺癌中的研究进展

**DOI:** 10.3779/j.issn.1009-3419.2017.09.07

**Published:** 2017-09-20

**Authors:** 悦超 刘, 影 邢, 莉 蔡

**Affiliations:** 150081 哈尔滨，哈尔滨医科大学附属肿瘤医院内四科 The Fourth Department of Medical Oncology, Harbin Medical University Cancer Hospital, Harbin 150081, China

**Keywords:** Hippo信号通路, 肺肿瘤, 进展, Hippo signaling pathway, Lung neoplasms, Progression

## Abstract

肺癌是全世界范围内肿瘤相关性死亡的首要原因，每年死亡人数超过100万人，占全球癌症死亡人数的五分之一。虽然目前在手术、放化疗、靶向治疗、免疫治疗肺癌方面取得了一定进展，但患者的预后仍不理想。因此，亟待寻找评价预后的分子标志物和肺癌的治疗新靶点，为肺癌患者提供生存获益的有效方法。近年来，Hippo信号通路逐渐成为国内外肿瘤研究领域中新兴且热门的研究方向。Hippo信号通路激活时，其核心组件MST/MOB、LATS1/2等能抑制转录的共激活剂YAP/TAZ的转录，二者被磷酸化并滞留在细胞浆中，从而抑制肺癌的发生发展。因此Hippo信号通路在临床应用中的潜在价值也越来越受关注。本篇文章总结了Hippo信号通路核心组成元件及上下游调控因子在肺癌形成进展过程中的重要作用和分子机制，并对Hippo信号通路的研究前景进行展望。

## Abstract (en)

Lung cancer is the leading cause of cancer related mortality in the world, with more than 1 million deaths per year, accounting for about one fifth of all cancer deaths worldwide. Over the past years, lung cancer treatment has been based on surgery, radiation therapy, chemotherapy, targeted therapies, and immunotherapy, but the improvement is not very perfect. Therefore, it has become clear that additional therapeutic strategies are urgently required to provide an improved survival benefit for patients. In recent years, Hippo signaling pathway has become a popular direction in the field of cancer research. When the Hippo pathway is active, the core Hippo kinase, such as MST/MOB and LATS1/2, inhibit the two transcriptional co-activators, YAP/TAZ. And YAP/TAZ are phosphorylated and sequestered in the cytoplasm. Dysregulation of the Hippo pathway drives multiple aspects of lung tumor initiation and progression. Moreover, the potential value of this pathway is getting more and more prevalent in clinical application. In this review, we summarize the molecular mechanism and the core components, upstream or downstream targets of Hippo signaling pathway which contribute the formation of lung cancer and discuss the therapeutic potential of targeted strategies in lung cancer. Additionally, we highlight the prospect of research on Hippo signaling pathway in the future.

肺癌是癌症相关死亡的主要原因之一，据世界卫生组织称，肺癌已成为当前全世界范围内最常见的癌症，每年能导致约160万人死亡，肺癌主要起源于支气管粘膜上皮，其中约85%为非小细胞肺癌（non-small cell lung cancer, NSCLC），其余为小细胞肺癌（small cell lung cancer, SCLC）^[1]^。虽然当今医疗技术水平不断提高，肺癌的总体预后并无明显改善，5年生存率仍低于15%，即使早期接受根治术的患者，仍然存在极高的复发几率，因此为进一步改善肺癌预后，迫切需要确定探索更多高效的治疗靶点药物^[2]^。研究人员逐渐发现Hippo信号通路出现在肺癌形成进展过程中，本文旨在概括Hippo信号通路中核心效应因子及其上下游靶点的异常表达对肺癌细胞的增殖、自我更新及侵袭迁移所发挥的重要作用。

## 1 Hippo信号通路概述

Justice等^[3]^于1995年首次发表了果蝇中*Warts*（*Wts*）基因的相关研究结果，并提出该基因对器官形状与大小具有调控作用，自此人们逐渐开展对Hippo信号通路的实验研究。果蝇中，Hippo信号通路是一种阻碍细胞生长的抑制性信号通路，该通路的重要成员LATS及Wts可以调控细胞增殖、器官大小及稳态，逐渐人们对Hippo信号通路的功能和调控有了更深入的认知。当果蝇中Hippo信号通路激活时，丝/苏氨酸激酶使Hpo发生磷酸化，并与Sav、Mob形成复合物，使Mats进一步磷酸化，并激活丝/苏氨酸激酶Wts。活化的Wts发生磷酸化，同时使Yki磷酸化，并与细胞质中14-3-3蛋白结合，最终导致磷酸化的Yki无法易位到细胞核中^[4]^。因此，Hippo信号通路能够抑制Yki转录靶基因的表达，从而抑制Yki相关效应基因的转录。Yki具有调控细胞增殖和细胞周期的功能，因此Hippo信号通路对维持器官稳态发挥重要作用（图 1A）。

**1:**
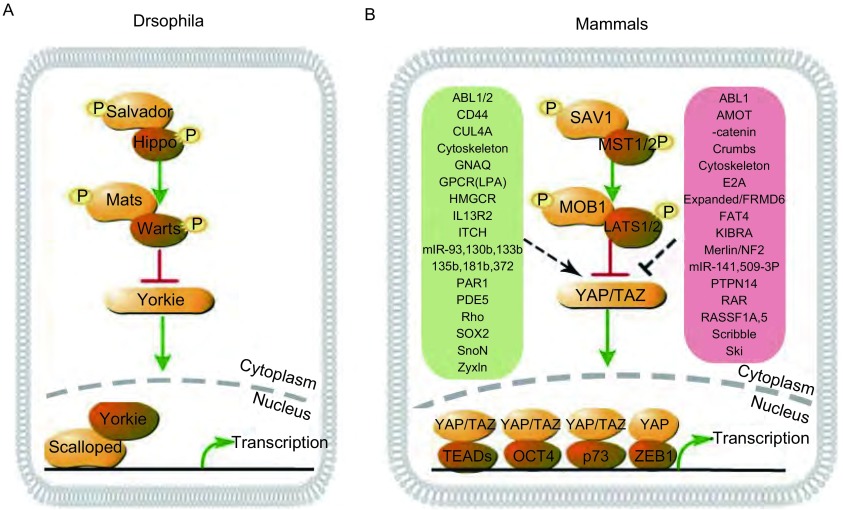
Hippo信号通路。A：果蝇中的Hippo信号通路；B：哺乳动物中的Hippo信号通路^[43]^。 Hippo signaling pathway. A: The Hippo signaling pathway in drosophila; B: The Hippo signaling pathway in mammalian^[43]^.

Hippo信号通路在进化中高度保守，在哺乳动物中，该通路的主要构成元件包含MST1/2、LATS1/2、YAP/TAZ等。Hippo信号通路激活后，MST1/2作为此激酶链的核心组件首先被激活，引起下游基因LATS1/2的磷酸化。LATS1/2主要通过阻滞细胞周期进程从而抑制肿瘤细胞的增殖迁移能力，并在介导细胞凋亡中发挥重要调控作用。LATS1/2作为YAP/TAZ的上游基因使二者发生磷酸化，因此磷酸化的YAP/TAZ增多，进而使易位到细胞核中的去磷酸化的YAP/TAZ量减少，与核内TEA域家族转录因子（TEA domain family members, TEAD）结合减少，抑制靶向基因转录，并最终抑制器官大小、肿瘤细胞增殖和转移等过程^[5]^。YAP/TAZ能够激活多种转录因子，包括TEAD家族成员、OCT4、p73及ZEB1等^[6]^（图 1B）。

近年来，针对Hippo信号通路在肿瘤发生发展中的研究逐渐深入，文献^[7-9]^报道Hippo信号通路参与肺癌、胆管癌、结肠癌、胰腺癌等多种肿瘤的进展过程。Lamar等^[10]^发现乳腺癌及黑色素瘤中YAP的过表达，可导致肿瘤细胞迁移能力增强、中心型黏附下降、出现间质表型并启动上皮-间质转化（epithelial-mesenchymal transition, EMT）过程。

## 2 Hippo信号通路在肺癌中的作用

### 2.1 Hippo信号通路的核心组件与肺癌

#### 2.1.1 MST/MOB

MST1（mammalian STE20-like kinase 1）能够编码丝氨酸苏氨酸激酶，是59 kDa的Ⅱ类激酶，其氨基酸序列与MST2具有76%的同源性，具有肿瘤抑制功能，二者的过表达可抑制NSCLC细胞的增殖、促进细胞凋亡^[11]^。通过体内及体外实验证实，人类NSCLC细胞系A549转染*MST1*基因的重组真核表达载体后，MST1的过表达促使YAP发生磷酸化，抑制细胞增殖，并诱导A549细胞发生凋亡^[11]^。而在另一种NSCLC细胞系H1299中敲除*MST1*基因后，促使突变型p53获得抗凋亡功能，证实*MST1*基因可以抑制肺癌细胞发生增殖^[12]^。Hippo信号通路另一个核心组件MOB（Mps one binder kinase）的mRNA表达在63%的NSCLC患者中明显降低，而在成年小鼠肺中MOB的敲除将导致细支气管细胞和细支气管肺泡干细胞（bronchioalveolar stem cells, BASCs）从基底膜脱离，有效抑制肿瘤发生；敲除*MOB*基因的转基因小鼠的支气管上皮细胞中，获得干性的细胞数目增加，通过对YAP/TAZ以及效应基因*NKX2.1*的调控，可促进支气管上皮细胞分化及肿瘤的形成^[13]^。因此，MST/MOB作为Hippo信号通路的核心组件对于抑制肺癌生长具有重要价值。

#### 2.1.2 LATS1/2

除MST/MOB外，Hippo信号通路的构成元件LATS1/2是肺癌发生发展过程中重要的肿瘤抑制因子。LATS1/2是Dbf2相关的核蛋白激酶家族成员，与Hippo信号通路在果蝇中的Wts属于同源物。Malik等^[14]^发现，LATS1过表达能够抑制裸鼠NSCLC细胞的增殖、非贴壁生长及肿瘤形成。人类肺癌细胞H460细胞中LATS1的表达上调了BAX蛋白水平从而促进肿瘤细胞凋亡，而用小干扰RNA敲除NSCLC细胞中的*LATS1*基因可明显增强细胞增殖及迁移能力；LATS1的同源异构体LATS2对肿瘤细胞同样具有抑制增殖及迁移的功能^[15]^。在临床肺癌组织样本研究中，LATS1在大多数NSCLC癌症患者中呈现低表达状态，并且其低表达与临床TNM分期、淋巴结转移及患者生存率紧密相关；大多数NSCLC患者中LATS2表达也降低，且突变比例不到10%，经*Cox*单因素和多因素回归分析，LATS2的表达水平是肺癌患者预后的独立预测因子^[16]^。此外，Moroishi等^[17]^研究证实LATS1/2激酶在肿瘤免疫过程中也具有阻碍肿瘤生长的作用，在宿主抗肿瘤免疫反应的诱导下，肿瘤抑制因子LATS1/2发生失活使肿瘤的免疫能力增强，证明Hippo信号通路能够调节肿瘤免疫原性，从而揭示在癌症免疫治疗中靶向针对LATS1/2位点将有效提高肿瘤治疗效果。

#### 2.1.3 YAP/TAZ

Hippo信号通路的效应分子YAP、TAZ均为肺癌的致癌基因，二者均定位于人类11q22染色体上，并在NSCLC中呈过表达。有70%的NSCLC细胞系中存在TAZ过表达，在正常支气管上皮细胞HBE135细胞中，TAZ过表达能够使非致瘤性的上皮细胞转化为高致瘤性细胞；而敲除TAZ后，效应基因*CTGF*及细胞周期调控蛋白Cyclin A表达明显降低，细胞周期停滞在G_0_期-G_1_期，抑制了NSCLC细胞体外非贴壁生长能力及体内的成瘤能力^[18]^。相似的结果也在TAZ的同源异构体YAP中发现，YAP的激活能够加速细胞增殖、抑制细胞凋亡，导致细胞接触性抑制丧失并促进细胞恶性转化，有效推进小鼠体内肺癌进展过程^[19]^。YAP/TAZ的过量表达使肺癌细胞迁移能力增强，并诱导其发生EMT过程，小鼠体内模型也证实YAP/TAZ的激活能够促进肺癌形成及转移过程的发生^[20]^。除此之外，超过60%的NSCLC患者存在*TAZ*基因过表达，且与低分化、淋巴结及远端转移、不良预后相关；60%-70%的NSCLC患者存在YAP过表达，并与临床TNM分期、淋巴结转移及患者生存率密切相关^[21]^。肺腺癌患者YAP的表达水平比肺鳞癌患者高，这是由于YAP受LKB1激活后可抑制肺腺癌细胞向鳞状细胞转化。

总之，Hippo信号通路的核心组件MST/MOB、LATS1/2、YAP/TAZ等均为肿瘤抑制因子或致癌因子，且在肺癌形成过程中发挥重要角色，参与调控肺癌进展的多步骤复杂过程。

### 2.2 Hippo信号通路的上游调节因子与肺癌

#### 2.2.1 LKB1

肝脏激酶B1（liver kinase B1, LKB1）是一种丝/苏氨酸激酶，具有肿瘤抑制作用。LKB1能够调节能量代谢、细胞极性和增殖，从而促进肺癌的进展。研究^[22]^证实，LKB1是Hippo信号通路核心组件YAP的重要上游调节因子。LKB1能够通过MARKScrib信号通路激活Hippo信号通路，导致YAP蛋白失活，最终抑制肺癌细胞生长，而敲除YAP后，抑制了LKB1缺失所诱导的肺癌细胞成瘤能力^[22]^。随后实验表明，YAP能特异性地被LKB1缺失所激活，抑制ZEB2依赖的DNp63表达，进而抑制鳞癌向腺癌的转化^[23]^。有研究称由于*LKB1*本身是一个抑癌基因，并不是理想的药物靶点，因此其下游蛋白逐渐成为肺癌治疗的潜在靶点，LKB1作为Hippo信号通路的关键调节因子，可以抑制肺癌的进展^[24]^。

#### 2.2.2 RASSF1A

RASSF1A（Ras-associated domain family 1A）是RAS相关结构域家族支架蛋白的主要成员之一，通过调节细胞周期进程和细胞凋亡发挥肿瘤抑制作用^[25]^。现已明确抑癌基因*RASSF1A*在肺癌中表达失活是由于启动子区CpG岛的特异性高甲基化所致^[26]^。30%-50%的NSCLC患者中可检测到RASSF1A的表达减少，并且*RASSF1A*甲基化是NSCLC术后患者不良预后的独立预测因素。RASSF1A作为Hippo信号通路关键的上游调节因子，可在DNA损伤后由ATM激活，随后通过激活MST2而诱导肿瘤细胞死亡^[25]^。在DNA复制过程中，RASSF1A可被ATR激酶激活，进而激活LATS1-Cdk2-BRCA2信号通路以维持基因组稳定性，而ATR-RASSF1A-MST2-LATS1信号轴的干扰会造成基因组缺陷，导致肺癌细胞基因组稳定性减弱、促进肿瘤发生^[27]^。因此，在肺癌的发生及进展过程中，RASSF1A在Hippo信号通路调控细胞凋亡及基因组失去稳定性方面至关重要。

#### 2.2.3 microRNAs

microRNAs（miRNA）是小非编码RNA（19个-22个核苷酸），mRNA的3'-UTR特异性结合靶蛋白导致靶基因蛋白表达降低。研究^[28]^指出，miRNA既具有致癌基因也具有肿瘤抑制基因的作用，同时参与肺癌的形成及转移过程。

miR-31是在肺癌中呈现过表达的致癌基因，敲除miR-31会抑制肺癌细胞生长和成瘤能力，并且miR-31可使小鼠及人类肺癌组织中LATS2表达减少，引起肺部肿瘤发生。肺转移的miRNA筛选研究发现，miR-135b具有抑制Hippo信号通路的核心成分LATS2和MOB1表达的特点，从而使NSCLC细胞表现为高侵袭性，在miR-135b过表达的NSCLC细胞中敲除TAZ，将明显降低癌细胞侵袭和集落形成能力^[29]^。在NSCLC患者样本中，miR-135b过表达与LATS2、核中TAZ的低表达及患者较差的生存率密切相关^[29]^。总之，上调miR-31和miR-135b可通过抑制Hippo信号通路，进一步促进肺部肿瘤发生和进展。除此之外，近来研究发现Hippo信号通路中的YAP/TAZ能够上调miR-25、miR-93、miR-106b的表达，最终抑制*p21*基因表达并促进肺癌进展^[30]^。而miR-335通过增强多药耐药过程，激活Hippo信号通路，最终抑制肺癌发展^[31]^。

### 2.3 Hippo信号通路的下游调节因子与肺癌

#### 2.3.1 LATS1/2的下游靶点

抑癌基因*p53*、凋亡蛋白Bcl2和Bcl-XL等为LATS1/2的下游靶点，这些基因可以通过抑制细胞周期调节剂Cdk1/细胞周期蛋白B（Cdk1/Cyclin B）及Cdk2/细胞周期蛋白E（Cdk2/Cyclin E）两种复合物，诱导肺癌细胞发生凋亡，抑制肺癌细胞增殖^[32]^。另外，LATS1可通过调控下游靶点Cdk2，促使BRCA2磷酸化、促进基因组的稳定性，抑制肺癌的发展^[33]^。然而，LATS1/2如何调节p53、Bcl2和Bcl-XL的机制尚不明确。

#### 2.3.2 YAP/TAZ的下游靶点

TTF1即NKX2-1，作为Hippo信号通路中YAP/TAZ的下游靶点，调节正常肺组织生长^[31]^。与TAZ及YAP类似，TTF1也具有致癌基因功能，并在肺腺癌中过表达。TAZ能够直接结合并激活TTF-1，进而激活下游靶点，如肺上皮细胞中的表面活性蛋白C（surfactant protein C, SP-C）靶点^[34]^。这些研究阐明YAP/TAZ与转录因子TTF1相互作用，在肺肿瘤发生过程中调节下游效应因子的转录，从而调控肺癌的恶性行为。除此之外，AXL、Cyr61、AREG和EPR均已被证实能够参与YAP/TAZ诱导的肺部肿瘤发生和转移^[35]^。

## 3 Hippo信号通路中的药物靶点

针对Hippo信号传导通路核心因子所研发的新兴肿瘤药物，如MST1/2小分子抑制剂（XMU-MP-1）、YAP靶点药物（维替泊芬）等药物的发现，对肿瘤的靶向治疗具有重要作用，为提高肺癌存活率提供新的治疗策略。

已有基础研究为Hippo信号通路的临床前和临床应用提供了理论基础，研究发现MST1/2小分子抑制剂（XMU-MP-1）能够选择性地结合MST1/2位点并抑制其表达，从而使YAP的表达水平增高，促进小鼠肝脏修复，在肝脏再生模型中发挥重要功效，进而使针对MST1/2靶点的特异性药物XMU-MP-1可能成为再生医学研究及组织修复的新型治疗手段^[36]^。研究证实，给予子宫内膜癌HEC-1-B细胞以维替泊芬处理后，免疫荧光染色及Western blot实验均证明细胞核中YAP表达明显下调，HEC-1-B细胞存活率显著降低，肿瘤的增殖受到抑制^[37]^。维替泊芬能通过抑制YAP及NF2的表达从而缩短视网膜母细胞瘤细胞的生存时间，证实维替泊芬通过阻断YAP-TEADs间相互作用，以达到抑制肿瘤的治疗目的^[38]^。Jiao等^[39]^模拟YAP的天然拮抗剂VGLL4的结构，设计出一种超级-TDU多肽，能与YAP竞争性结合TEAD位点以抑制YAP的表达，并在胃癌动物模型及MGC-803等胃癌细胞系中证实超级-TDU多肽干扰YAP-TEADs复合物的形成能力使YAP-TEADs作用的下游靶基因*CTGF*、*CYR61*及*CDX2*的表达发生下调，进而抑制胃癌细胞的增殖能力，减小裸鼠胃癌肿瘤的大小，因此超级-TDU多肽对YAP相关的恶性肿瘤具有重要的治疗作用。除此之外，维替泊芬通过特异性地阻止易位入核的YAP与TEAD位点结合，增强了肺癌细胞系H1975细胞对靶向药物厄洛替尼的敏感性^[40]^，为临床应用中逆转厄洛替尼耐药提供新方法。目前随着YAP/TAZ诱导NSCLC细胞上皮-间质转化、促进转移、促进干细胞特性获得、调控凋亡等相关研究逐渐深入^[19]^，设计针对Hippo信号通路的分子靶向药物，必将为肺癌转移的分子诊断和精准治疗提供新的视角，也使Hippo信号通路具有重要的临床意义。

## 4 总结与展望

Hippo信号通路由多种肿瘤抑制因子构成激酶链，调控肿瘤细胞增殖和凋亡之间的动态平衡，以达到抑癌的最终目的。本篇综述总结了Hippo信号通路的核心组件及其上下游调节因子在肺癌形成及进展中的重要作用。此外，Hippo信号通路在形成肺癌干细胞过程中发挥重要作用，这对于未来探究Hippo信号通路如何参与肺癌干细胞发育过程具有指导意义^[41]^。而后，Hippo信号通路与Wnt、Notch等多条信号转导通路之间存在相互作用关系，目前已发现Wnt通路与肺动态平衡紧密相关^[42]^，因此进一步探索Hippo信号通路与这些信号通路相互作用关系及如何调节肺发育、肿瘤发生及转移的分子机制尤为必要。

同时仍有许多问题有待进一步探究，如肺癌晚期Hippo信号通路与肿瘤微环境的相互作用、解决肺癌治疗过程中的耐药问题等等。未来Hippo信号通路有望对改进当前肺癌的治疗方案产生更加深远的影响，它在癌症中所发挥的重要角色使我们开始思考，如何把Hippo信号通路应用成为阻滞肺癌进展的有效方法和有利手段，设计临床新靶点治疗药物，从而为临床上肺癌的诊断、治疗及防治开辟新思路。
